# An Arthropod Enzyme, Dfurin 1, and a Vertebrate Furin Homolog Display Distinct Cleavage Site Sequence Preferences for a Shared Viral Proprotein Substrate

**DOI:** 10.1673/031.010.2901

**Published:** 2010-04-04

**Authors:** Gina L. Cano-Monreal, Jacqueline C. Williams, Hans W. Heidner

**Affiliations:** Department of Biology, The University of Texas at San Antonio, San Antonio, Texas 78249-0662.

**Keywords:** *Aedes aegypti*, *Aedes albopictus*, alphavirus, Chinese hamster, *Cricetulus griseus*, *Drosophila melanogaster*, Proprotein convertase, Sindbis virus, *Togavirdae*

## Abstract

Alphaviruses replicate in vertebrate and arthropod cells and utilize a cellular enzyme called furin to process the PE2 glycoprotein precursor during virus replication in both cell types. Furin cleaves PE2 at a site immediately following a highly conserved four residue cleavage signal. Prior studies demonstrated that the amino acid immediately adjacent to the cleavage site influenced PE2 cleavage differently in vertebrate and mosquito cells (HW Heidner et al. 1996. Journal of Virology 70: 2069–2073.). This finding was tentatively attributed to potential differences in the substrate specificities of the vertebrate and arthropod furin enzymes or to differences in the carbohydrate processing phenotypes of arthropod and vertebrate cells. To further address this issue, we evaluated Sindbis virus replication and PE2 cleavage in the Chinese hamster, *Cricetulus griseus* Milne-Edwards (Rodentia: Cricetidae) ovary cells (CHO-K1) and in a CHO-K1-derived furin-negative cell line (RPE.40) engineered to stably express the Dfurin1 enzyme of *Drosophila melanogaster* Meigen (Diptera: Drosophilidae). Expression of Dfurin1 enhanced Sindbis virus titers in RPE.40 cells by a factor of 10^2^ – 10^3^, and this increase correlated with efficient cleavage of PE2. The PE2-cleavage phenotypes of viruses containing different amino acid substitutions adjacent to the furin cleavage site were compared in mosquito (C6/36), CHO-K1, and Dfurin1-expressing RPE.40 cells. This analysis confirmed that the substrate specificities of Dfurin1 and the putative mosquito furin homolog present in C6/36 cells are similar and suggested that the alternative PE2 cleavage phenotypes observed in vertebrate and arthropod cells were due to differences in substrate specificity between the arthropod and vertebrate furin enzymes and not to differences in host cell glycoprotein processing pathways.

## Introduction

Many viruses express glycoproteins in the form of an inactive precursor and then utilize furin, a host-cell enzyme,to cleave the precursor into its mature functional form (Garten et al. 1994). Furin localizes primarily to the trans-Golgi network and the plasma membrane; thus, viral proproteins are cleaved by furin during their transit through the trans-Golgi or after arrival at the cell surface (Thomas 2002). Furin cleaves proproteins at a site immediately following four residues ordered in a basic-X-basic-basic (bxbb) motif (Hosaka et al. 1991). Although the bxbb motif represents the core furin cleavage signal, the residue immediately adjacent to this motif (+1 position) influences furin-mediated cleavage of a range of viral proprotein substrates in vertebrate cells (Toyoda et al. 1987; Morrison et al. 1993; Heidner and Johnston 1994; Horimoto and Kawaoka 1995; Fujii et al. 1999). Specifically, cleavage of the viral proproteins is strongly inhibited in vertebrate cells when residues at the +1 position contain branched aliphatic side chains (isoleucine, leucine, valine). Furin-like enzymes produced by insect cells also recognize the consensus bxbb motif (De Bie et al. 1995; Chen et al. 1996; Cieplik et al. 1998). It is not known if residues at the +1 position influence insectderived furin enzymes' cleavage of proproteins.

Alphaviruses are relatively simple, enveloped, positive strand RNA viruses that belong to the *Togaviridae* virus family. Alphaviruses have a global distribution and many members of this genus cause significant disease in humans, ranging from fever, arthralgia and rash, to lethal encephalitis (Griffin DA 2001). Sindbis virus (Group IV: Togaviridae: alphavirus) is the prototype member of the genus, and its structural and biological properties have been studied extensively (for review: Strauss and Strauss 1994). The natural alphavirus maintenance cycle involves alternating infections of vertebrate and arthropod hosts with mosquitoes serving as the most common insect vector. The alphavirus glycoproteins are synthesized as components of a polyprotein that is cleaved into proteins designated as PE2, 6K, and E1 by the host cell signal peptidase enzyme in the rough endoplasmic reticulum (Strauss and Strauss 1994). PE2 and El proteins associate into PE2/E1 heterodimers and then are processed and transported through the exocytic pathway of the host cell (Erwin and Brown 1980; Rice and Strauss 1982). The PE2 protein is cleaved by a host cell endoprotease as the heterodimers are transported through a trans or post-Golgi compartment (Jones et al. 1974; De Curtis and Simmons 1988). The major product of PE2 cleavage is the E2 glycoprotein which, together with E1, forms the glycoprotein spikes that project from the surface of mature virions. Cleavage of PE2 occurs immediately downstream of a bxbb motif (Rice and Strauss 1981). Furin has been shown to mediate PE2 cleavage during alphavirus replication in vertebrate cells (Watson et al. 1991; Moehring et al. 1993; Zhang et al. 2003; Ozden et al. 2008). The cellular enzyme that cleaves PE2 in mosquito cells has not been identified but is likely to be an arthropod homolog of the vertebrate furin enzyme, as mutations that block access to the bxbb sequence and restrict PE2 cleavage in vertebrate cells also restrict PE2 cleavage and virus replication in mosquito cells (Presley et al. 1991; Heidner et al. 1996). In addition, deletion of the bxbb sequence prevents PE2 cleavage in vertebrate cells and restricts virus replication in cultured mosquito cells and within living mosquitoes (Davis et al. 1995; Turell et al. 1999).

Cleavage of PE2 in vertebrate cells is profoundly influenced by the residue immediately following the bxbb motif (Heidner and Johnston 1994). Specifically, cleavage of PE2 proteins containing isoleucine, valine, or leucine was greatly reduced compared to PE2 substrates with other amino acids at the +1 position. Interestingly, when Sindbis virus variants containing valine or leucine were grown in cultured mosquito cells, PE2 was cleaved efficiently and the virus replicated with normal kinetics (Heidner et al. 1996). The distinct PE2 cleavage phenotypes in vertebrate and mosquito cells could result from differences in the substrate specificities of the vertebrate and arthropod furin enzymes. Alternatively, they could result from differences in the carbohydrate-processing phenotypes of arthropod and vertebrate cells, which could differentially influence access of the furin enzyme to the PE2 cleavage site. Specifically, the N-linked oligosaccharides synthesized in vertebrate cells typically consist of complex or hybrid structures (Kornfeld and Kornfeld 1985). In contrast, Nlinked oligosaccharides synthesized in insect cells are typically restricted to low-mannose and high mannose forms (März et al. 1995). Consequently, the structures of N-linked oligosaccharides on alphavirus glycoproteins differ markedly depending on the host cell (vertebrate vs. arthropod) used to propagate virus, and these host-specific differences can have a profound influence on the biological properties of the virus (Hsieh et al. 1983; Boehme et al. 2000a; Boehme et al. 2000b; Klimstra et al. 2003). The objective of this study was to differentiate between these two alternatives.

Virus replication and PE2 cleavage phenotypes were compared in a Chinese hamster ovary cell line (CHO-K1) and in a CHO-K1-derived furin-negative cell line (RPE-40) engineered to express the Dfurin1 enzyme from *Drosophila melanogaster* Meigen (Diptera: Drosophilidae). The use of Dfurin1 was based on the evolutionary relatedness of mosquitoes (Diptera: Culicidae) and *D. melanogaster* and on the likelihood that the furin enzymes derived from both flies share similar genetic and functional properties. Dfurin1 is expressed abundantly in adult flies and correctly processes substrates containing the consensus sequence for mammalian furin enzymes (Roebroek et al. 1993; De Bie et al. 1995). This cell culture system made it possible to evaluate and compare the substrate specificities of representative vertebrate and arthropod furin enzymes under conditions where the glycosylation properties of the PE2 substrate remained constant.

## Materials and Methods

### Viruses and cells

The parental Sindbis virus (strain AR339), TRSB, has been described previously (McKnight et al. 1996). The genetic and phenotypic properties of the TRSB-derived mutant viruses, TRSB-NE2G216, TRSB-E2S1, TRSB-E2L1, TRSB-E2V1, TRSB-E2F1, TRSB-E2N1, TRSB-E2D1, and TRSB-E2H1 also have been described (Heidner and Johnston 1994; Heidner et al. 1996). In this report, the mutant viruses are referred to as NE2G216, E2S1, E2L1, E2V1, E2F1, E2N1, E2D1, andE2H1, respectively.

BHK-21 cells were obtained from the American Type Culture Collection. The CHOK1 and RPE.40 cell lines have been described (Moehring and Moehring 1983; Spence et al. 1995). BHK-21, CHO-K1 and RPE.40 cells were maintained at 37° C in alpha minimum essential medium supplemented with 10% donor calf serum, 10% tryptose phosphate broth, and antibiotics. C6/36 cells were originally derived from *Aedes albopictus* Skuse (Diptera: Culicidae) larvae (Igarashi 1978). C6/36 cells were maintained at 28° C in alpha minimal essential medium supplemented with 10% fetal calf serum, 10% tryptose phosphate broth, and antibiotics.

### Plasmid constructions

Construction of a vector for stable expression of Dfurin1 in eukaryotic cells required the use of several shuttle vectors. The cDNA sequences of Dfurin1 were derived from a phagemid designated pIP63 (Roebroek et al. 1993). First, the entire Dfurin1 sequence was amplified by PCR using pIP63 DNA as template and oligonucleotide primers that incorporated an XbaI restriction site (5′) and an ApaI restriction site (3′). The amplicon product was digested with XbaI and ApaI and ligated into a plasmid called SINrep5 (Bredenbeek et al. 1993) from which a corresponding XbaI/ApaI fragment had been removed. The resulting construct was designated pSIN-Dfur1. Second, the 549 5′ terminal base pairs of the Dfurin1 gene were amplified by PCR using pIP63 DNA as template and oligonucleotide primers that incorporated an XbaI restriction site and a consensus Kozak translation initiation sequence (5′), and which flanked a unique BamHI restriction site at nucleotide 549 of the Dfurin1 coding sequence (3′). The PCR product was digested with XbaI and BamHI and ligated into a plasmid designated pH3′2J1 (Hahn et al. 1992) from which a corresponding Xbai/BamHI fragment had been removed, to produce pH3/Dfur5′. Third, the remaining Dfurin1 sequences were transferred from pSIN-Dfur1 by subcloning of a BamHI/XhoI fragment into pH3/Dfur5′ to produce pH3/Dfur1. Finally, the entire Dfurin1 coding sequence was subcloned from pH3/Dfur1 into the eukaryotic expression vector pcDNA3.1 (Invitrogen, www.invitrogen.com) by transfer of a XbaI/NotI fragment. This transfer placed the Dfurin1 coding sequences under transcriptional control of the human cytomegalovirus immediate early promoter. The resulting construct, pc3.1/Dfur1 was sequenced across the entire Dfurin1 region to confirm the sequence of the DNA insert.

### Generation of stably transformed RPE.40 cells

Plasmid DNAs were purified by use of the Wizard Purefection Kit (Promega, www.promega.com). RPE.40 cells were transformed with the pcDNA3.1 control plasmid or with the pc3.1/Dfur1 plasmid using the Calcium Phosphate Transfection Kit (Invitrogen). pcDNA3.1-based plasmids confer neomycin resistance, thus, cultures of stably transformed cells were selected in the presence of G418 sulfate (400 µg/ml) over a two week period. Cells were plated onto 150 mm culture dishes at a low density and individual cells were isolated using sterile cloning rings. Isolated cells were then expanded into clonal cell lines under constant G418 sulfate selection. In all subsequent experiments using transformed RPE.40 cell lines, G418 sulfate was included in the growth medium at a concentration of 400 µg/ml.

### Indirect immunofluorescence staining of cells

CHO-K1, RPE.40, and transformed RPE.40 cell lines were grown to approximately 50% confluence in 8-well chamber slides. Cells were fixed in 4% paraformaldehyde (in PBS), and permeabilized with 0.1% Triton X-100 (in PBS). Cells were blocked with 10% bovine serum albumin (in PBS) and then probed for 1 hour with a non-immune rabbit serum or with a polyclonal rabbit antiserum raised against a Dfurin1/glutathione S-transferase fusion protein diluted in 25% donor calf serum (in PBS). Cells were washed extensively with 10 mM glycine/.05% Tween-20 in PBS and then probed with fluorescein isothiocyanate-conjugated goat anti-rabbit IgG secondary antibody (Sigma-Aldrich, www.sigmaaldrich.com). Cells were incubated for 1 hour, washed with 10 mM glycine/0.05% Tween-20 in PBS. Cells were then treated with 4′,6-diamidino-2-phenylindole (DAPI) (10 µ g/mL, Sigma-Aldrich) to stain nuclei and then analyzed by fluorescence microscopy (Axioskop, Carl Zeiss, www.zeiss.com).

### Kinetics of viral growth in transformed and non-transformed cell lines

The kinetics of virus growth was determined for various viruses in CHO-K1, RPE.40, and Dfurin1-transformed RPE.40 cell lines. Infections were performed on duplicate monolayers of cells grown in 60 mm Petri dishes (2 × 10^6^ cells/dish) and were initiated by infection with free virus at a multiplicity of infection of 10 plaque forming units (pfu) per cell. Virus was adsorbed to cells for 30 minutes, and, then, remaining virions were removed by repeated washes. Cells were overlaid with medium and maintained at 37° C. Supernatant samples were collected at regular intervals post-infection, clarified by microcentrifugation, and stored at -70° C. Infectious virus in each sample was quantified by plaque assay on BHK-21 cells. Virus titers are reported here as the average of the duplicate samples.

### Polyacrylamide gel analysis of [^35^S]methionine-labeled viral proteins

Virions were metabolically radiolabeled with [^35^S]-methionine during growth in CHO-K1, RPE.40, transformed RPE.40 cells lines, and C6/36 cells as described (Heidner et al. 1994). Radiolabelled virions were purified from cell supernatants by isolation on discontinuous potassium tartrate gradients (20% / 35%) followed by banding on continuous potassium tartrate gradients (20% to 35%). Potassium tartrate solutions were made in TNE buffer (0.5 M Tris-HCl (pH 7.2), 0.1 M NaCl, and 0.001 M EDTA). Banded virions were collected and pelleted through sucrose cushions (20% in TNE) by ultracentrifugation. Due to the instability of PE2-containing virions derived from RPE.40 cells, viruses were purified by a simple pelleting technique in which infected cell supernatants were clarified of cell debris by high speed spin, passed through a .45 micron filter, and then pelleted by ultracentrifugation. The virus pellet was then washed once with TNE buffer, re-pelleted by ultracentrifugation, and harvested. Radiolabelled virion preparations were quantified by liquid scintillation counting and equal quantities of each were resolved by SDS-PAGE (10% acrylamide) prepared as described (Laemmli 1970).

### Comparison of Dfurin1 with mosquito-derived proprotein convertase enzymes

To identify furin homologs in the *Aedes aegypti* L.(Diptera: Culicidae) mosquito, a BLASTP search was performed with the default parameter setting, using the protein sequence of *D. melanogaster* Dfur1 (accession number AAA28549) as a query sequence against the VectorBase (http://www.vectorbase.org/index.php). Conserved domains/motifs were identified by searching the Pfam protein family database (Finn et al. 2008). Multiple alignments were generated using the T-coffee program (Notredame et al. 2000), followed by manual inspection and editing.

## Results

### Generation of RPE.40 cells stably expressing Dfurin1

**Figure 1.**
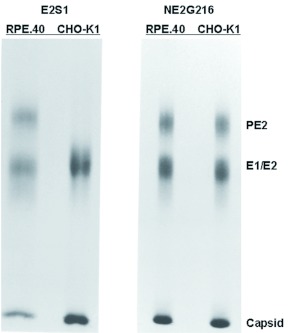
SDS-PAGE analysis of purified E2S1 and NE2G216 virions grown in furin-negative RPE.40 cells and in the furin-positive parental cell line CHO-K1. Virions were metabolically radiolabeled with [^35^S]-methionine during growth in each cell line and purified from cell supernatants as described in the text. High quality figures are available online.

RPE.40 cells were permissive for Sindbis virus replication but failed to cleave PE2 due to genetic mutations within both furin alleles (Watson et al. 1991; Spence et al. 1995). These phenotypes were confirmed by comparing the PE2 cleavage phenotypes of two Sindbis viruses, E2S1 and NE2G216, during growth in CHO-K1 and RPE.40 cells. E2S1 was used in place of TRSB because it encodes a PE2 glycoprotein with an optimal cleavage site for the furin enzyme expressed in cultured hamster cells (Heidner and Johnston 1994; Klimstra et al. 1999). As predicted, E2S1 virions derived from CHOK1 cells contained E2 and E1 glycoproteins, and virions derived from RPE.40 cells contained uncleaved PE2 and E1 (Figure 1). NE2G216 is defective for PE2 cleavage in all cell types due to the placement of an N-linked oligosaccharide adjacent to the furin cleavage site (Heidner et al. 1994), and NE2G216 virions retained PE2 in place of E2 when grown in both cell types (Figure 1).

**Figure 2.**
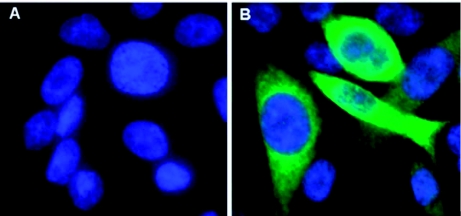
Expression of Dfurin1 in transformed RPE.40 cells. Parental RPE.40 cells (A), and the cell line R-Dfur1#11 (B), were analyzed for Dfurin1 expression using an indirect immunofluorescence assay. Permeabilized cells were probed with a polyclonal rabbit antiserum raised to a Dfurin1/glutathione S-transferase fusion protein and then probed with a FITC-conjugated goat anti-rabbit IgG secondary antibody. Cells were stained with DAPI and analyzed by fluorescence microscopy (magnification = 400×). High quality figures are available online.

RPE.40 cells were transfected with pc3.1/Dfur1 plasmid DNA or with the control plasmid pcDNA3.1, and stably transformed cells were isolated under G418 sulfate selection. Individual cells from the pc3.1/Dfur1 transfection were expanded into clonal cell lines (R-Dfur1); however, cells transformed with the control plasmid (R-3.1) were not cloned further. Based on the results obtained from pilot virus growth assays, cell lines R-Dfur1#11 and R-Dfur1#22 were selected for further study. To confirm that these cells expressed the Dfurin1 enzyme, CHO-K1, RPE.40, R-Dfur1#11 and RDfur1#22 cell lines were probed with nonimmune rabbit serum or with a polyclonal rabbit antiserum raised against a Dfurin1/glutathione S-transferase fusion protein in an indirect immunofluorescence assay. Staining was not observed in any cell line probed with the non-immune antiserum or in RPE.40 and CHO-K1 cells probed with the Dfurin1-specific antiserum (data not shown). In contrast, bright staining was observed in RDfur1#11 and R-Dfur1#22 cells probed with the Dfurin1-specific antiserum, but not in the parental RPE.40 cell line (Figure 2). Staining localized to the perinuclear region which is consistent with the Golgi-specific localization that is predicted for the Dfurin1 enzyme.

### The effects of Dfurin1-expression on Sindbis virus replication

**Figure 3.**
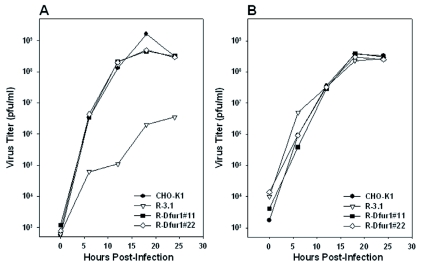
Kinetics of virus growth for wild-type virus, TRSB (A), and for the PE2-cleavage defective mutant virus, NE2G216 (B) in the CHO-KI, R-3.1, R-Dfur1#11 and R-Dfur1#22 cell lines. High quality figures are available online.

**Figure 4.**
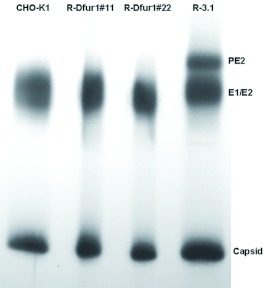
Determination of the PE2-cleavage phenotype for E2S1 grown in the CHO-K1, R-Dfur1#11, R-Dfur1#22, and R3.1 cell lines. Virions were metabolically radiolabeled with [^35^S]-methionine during growth in each cell line, purified from cell supernatants, and analyzed by SDS-PAGE as described in the text. High quality figures are available online.

The kinetics of viral growth were assessed in the CHO-K1, R-3.1, R-Dfur1#11 and RDfur1#22 cell lines following infection with TRSB or NE2G216. TRSB replicated to similar titers and with similar kinetics in the CHO-K1, R-Dfur1#11, and R-Dfur1#22 cell lines (Figure 3A). TRSB titers from these cell lines were 2–3 log_10_ higher than those produced in R-3.1 cells (Figure 3A). The reduced titer of TRSB grown in R-3.1 cells is consistent with previous reports, and has been shown to result from a decrease in virion infectivity associated with retention of PE2 in virions grown under these conditions, and not from decreased virus yield from these cells (Watson et al. 1991; Moehring et al. 1993; Heidner et al. 1994). As expected, expression of Dfurin1 in RPE.40 cells had no detectable effect on the growth of the PE2 cleavage-defective virus, NE2G216, which grew to comparable titers in all four cell lines (Figure 3B). NE2G216 contains a mutation at E2 residue 216 (glutamic acid to glycine) that facilitates normal replication of this virus in the absence of PE2 cleavage (Heidner et al. 1994). These results suggested that the increased titers of TRSB in the R-Dfur1#11 and R-Dfur1#22 cell lines was due to increased virion infectivity associated with Dfurin1-mediated cleavage of PE2 in these cells. To investigate PE2-cleavage in these cells, E2S1 virions were grown in each cell line and analyzed by SDS-PAGE (Figure 4). As predicted, PE2 was not cleaved during viral replication in R-3.1 cells, but was cleaved efficiently in the CHO-K1, R- Dfur1#11 andR-Dfur1#22 cell lines.

### Substrate specificities of vertebrate and arthropod furin enzymes

**Figure 5.**
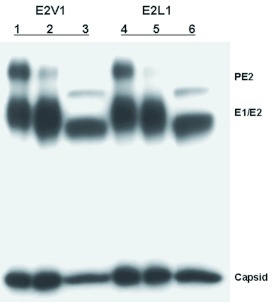
SDS-PAGE analysis of purified E2V1 (lanes 1–3) and E2L1 (lanes 4–6) virions grown in CHO-K1 (lanes 1 and 4), RDfur1#22 (lanes 2 and S), and C6/36 (lanes 3 and 6) cells. Virions were metabolically radiolabeled with [^35^S]-methionine during growth in each cell line, purified from cell supernatants, and analyzed by SDS-PAGE as described in the text. High quality figures are available online.

Generation of the Dfurin1-expressing RPE.40 cells made it possible to compare the substrate preferences of model vertebrate (*Cricetulus griseus* Milne-Edwards (Rodentia: Cricetidae)) and arthropod (*D. melanogaster*) furin enzymes under conditions that eliminated host-specific differences in glycoprotein processing. To accomplish this, the PE2 cleavage phenotype of TRSB and of seven TRSB-derived mutants was determined in the CHO-K1 and R-Dfur1#22 cell lines. In addition, PE2 cleavage was assessed following growth of these viruses in C6/36 cells. Each of the viruses contained a different amino acid at the +1 position. Viral proteins were radiolabeled during growth in each cell line, purified from cell supernatants, and analyzed by SDS-polyacrylamide gel electrophoresis. Essentially complete cleavage of PE2 was detected in all three cell lines when the infecting viruses contained arginine (TRSB), serine (E2S1), phenylalanine (E2F1), histidine (E2H1), asparagine (E2N1), or aspartic acid (E2D1) at the +1 position (data not shown). In contrast, obvious differences in PE2 cleavage efficiency were observed between the cell lines when the infecting viruses contained valine (E2V1) or leucine (E2L1) at the +1 position (Figure 5). Consistent with previous reports, PE2 substrates containing leucine or valine at the +1 position were cleaved much more efficiently, albeit not completely, by the arthropod enzymes than by the vertebrate enzyme (Figure 5). As expected, viral glycoproteins derived from C6/36 cells migrated faster than cognate viral glycoproteins derived from the vertebrate cells due to differences in their carbohydrate structures.

## Discussion

The objective of this study was to determine the basis for the alternative cleavage fates of select Sindbis virus PE2 substrates in cultured vertebrate and arthropod cells. By evaluating PE2 cleavage in CHO-K1 and Dfurin1 expressing RPE.40 cells, it was possible to compare the cleavage site preferences of a model vertebrate (*C. griseus*) and arthropod (*D. melanogaster*) furin enzyme under conditions where the glycosylation properties of the PE2 substrate remained constant. The results indicated that the alternative PE2 cleavage phenotypes were not linked to differences in the carbohydrate processing phenotypes between the two cell types. The study did establish that the vertebrate and arthropod enzymes are differentially influenced by the amino acid occupying the +1 position relative to the bxbb cleavage signal and that this difference accounts for the alternative cleavage fates of the PE2 proteins in vertebrate and arthropod cells. Specifically, PE2 proteins containing valine or leucine residues at the +1 position were largely resistant to cleavage by the vertebrate furin enzyme, but were cleaved efficiently (albeit not 100%) by Dfurin1. These same substrates were cleaved efficiently in cultured C6/36 cells (albeit not 100%) , which suggests that mosquito cells produce a furin-like enzyme with cleavage site preferences similar to Dfurin1.

**Figure 6.**
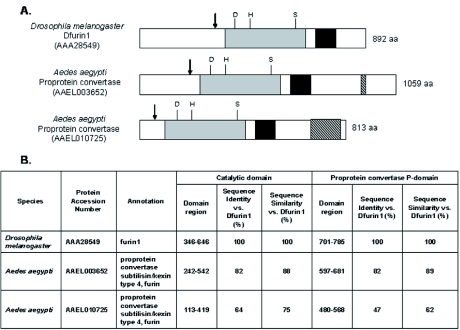
Comparisons of overall protein structure (A) and protein sequence within the catalytic and P domains (B) between the Dfurin1 protein of *Drosophila melanogaster* and the most closely related proprotein convertases of Aedes *aegypti*. (A) Schematic diagram of the three furin-like proprotein convertase enzymes showing approximate positions of the prodomain cleavage site (↓), subtilisin-like catalytic domain (shaded grey) including the positions of the active site residues (Asp, His, and Ser), the P domain (shaded black), and the cystein-rich domain (hatched area). (B) Sequence summary table showing sequence identity and sequence similarity between the catalytic and P domains of *Aedes aegypti* proprotein convertase enzymes and Dfurin1. High quality figures are available online.

It is generally accepted that PE2 is cleaved by a furin-like enzyme during virus replication in mosquito cells. However, to our knowledge, this study is the first to directly evaluate the ability of an arthropod-derived furin enzyme to support alphavirus replication through proteolytic processing of PE2. The vertebrate and arthropod furin-like enzymes belong to the proprotein convertase family within the subtilisin superfamily of serine proteases. The proprotein convertase enzymes share common structural features, including an N-terminal prodomain that is removed by autoproteolytic cleavage, a subtilisin-like catalytic domain with an active site composed of an aspartate, histidine, serine catalytic triad, a P domain which is essential for enzymatic activity, and, in some cases, a cystein-rich domain of unknown function near the C-terminus (Thomas 2002). During the completion of this project, the genomic sequence of the *A. aegypti* mosquito was completed and shown to encode several furin-like enzymes that were predicted to share these features (Nene et al. 2007). Two of these enzymes (accession numbers AAAEL003652 and AAEL010725) display a particularly high degree of sequence and predicted structural similarity to Dfurin1 (Figure 6). The most closely related of these (AAEL003652), shares 82% sequence identity with Dfurin1 within both the catalytic domain and the P domain (Figure 6). This enzyme is nearly identical to an *A. aegypti* enzyme previously identified as vitellogenin convertase (accession number AAC37262). It was cloned from a cDNA library, and the expression properties and enzymatic activities of the enzyme were characterized (Chen and Raikhel 1996). Vitellogenin convertase plays an important role in vitellogenesis (Chen and Raikhel 1996) by cleaving its pro-vitellogenin substrate downstream of a bxbb motif (RYRR↓D) (Sappington and Raikhel 1998). It is expressed to high levels in the fat body, and its expression is induced following the ingestion of a blood meal (Chen and Raikhel 1996). Vitellogenin convertase probably is capable of cleaving PE2 and, based on sequence considerations, is a good candidate for the PE2-processing enzyme in mosquitoes. However, it is not known if it is expressed in mosquito tissues relevant to Sindbis virus replication and transmission, such as the salivary gland, or if it plays a role in processing viral proproteins during natural infections of mosquitoes by alphaviruses or flaviviruses. The second furin-like proprotein convertase identified in the mosquito genome project (AAELO 10725) is predicted to share common proprotein convertase structural features with Dfurin 1, but displays a lower level of sequence identity (64% sequence identity with the catalytic domain and 47% identity within the P domain) (Figure 6). The tissue distribution and enzymatic properties of this protein have not been studied.

The cyclic nature of alphavirus replication places unique selective pressures on the virus as viral proteins and genetic elements must maintain their ability to functionally interact with the cellular components of both evolutionarily diverged hosts. As a consequence, alphaviruses are thought to evolve compromise genotypes that are not optimally adapted to either host (Greene et al. 2005). Indeed, repeated virus passage within a single cell type (vertebrate or arthropod) leads to the generation of host range mutants that display increased virus fitness in the cell type used for passage and a concomitant fitness decrease in the cell type that was bypassed (Weaver et al. 1999; Cooper and Scott 2001; Greene et al. 2005). Similar results were obtained when an alphavirus (Venezuelan equine encephalitis virus) was repeatedly passaged through mosquitoes or mice (Coffey et al. 2008). Results from this study suggested that the distinct substrate preferences of the arthropod and vertebrate furin enzymes would influence evolution of the residue immediately downstream of the PE2 cleavage site. A comparison of viral sequences revealed that most alphaviruses encode a serine residue at the +1 position (McKnight et al. 1996), and this residue appears to be optimal for furin cleavage in vertebrate cells (Heidner and Johnston 1994; Klimstra et al. 1999). Presumably, if alphaviruses containing valine or leucine at this position were to arise during the mosquito phase of the maintenance cycle, they would likely be selected against in the vertebrate host due to inefficient cleavage of PE2 by the vertebrate furin enzyme and the adverse effect that this phenotype has on viral replication in the vertebrate host (Heidner and Johnston 1994). Like the alphaviruses, members of the flavivirus genus (family *Flaviviridae*) utilize furin to cleave a glycoprotein precursor, prM, immediately downstream of a bxbb motif (Stadler et al. 1997). Serine also occupies the +1 position in the prM proprotein of nearly all insect vectored members of the flavivirus genus (Keelapang et al. 2004). Interestingly, the prM protein of Kamiti River virus contains alanine at the +1 position, and the *Culex* flavivirus contains valine at this site (Crabtree et al. 2003; Hoshino et al. 2007). Kamiti River virus and *Culex* flavivirus are insect-only flaviviruses and, therefore, are not subjected to any selective pressure in vertebrate cells.

## Abbreviations

bxbb: basic-X-basic-basic;
CHO: Chinese hamster ovary;
pfu: plaque forming unit
